# Effect of Feeding Cows with Unsaturated Fatty Acid Sources on Milk Production, Milk Composition, Milk Fatty Acid Profile, and Physicochemical and Sensory Characteristics of Ice Cream

**DOI:** 10.3390/ani9080568

**Published:** 2019-08-17

**Authors:** Einar Vargas-Bello-Pérez, Nathaly Cancino-Padilla, Carolina Geldsetzer-Mendoza, Stefanie Vyhmeister, María Sol Morales, Heidi Leskinen, Jaime Romero, Philip C. Garnsworthy, Rodrigo A. Ibáñez

**Affiliations:** 1 Departamento de Ciencias Animales, Facultad de Agronomía e Ingeniería Forestal, Pontificia Universidad Católica de Chile, Casilla-306 Santiago, Chile; 2 Department of Veterinary and Animal Sciences, Faculty of Health and Medical Sciences, University of Copenhagen, Grønnegårdsvej 3, DK-1870 Frederiksberg C, Denmark; 3 Departamento de Fomento de la Producción Animal, Facultad de Ciencias Veterinarias y Pecuarias, Universidad de Chile, Av. Santa Rosa 11735, La Pintana, Chile; 4 Milk Production, Production Systems, Natural Resources Institute Finland (Luke), FI-31600 Jokioinen, Finland; 5 Laboratorio de Biotecnología de Alimentos, Unidad de Alimentos, Instituto de Nutrición y Tecnología de los Alimentos (INTA), Universidad de Chile, Santiago 7830490, Chile; 6 School of Biosciences, The University of Nottingham, Sutton Bonington Campus, Loughborough LE12 5RD, UK

**Keywords:** bioactive compounds, dairy products, fatty acids, fish oil, sensory properties, soybean oil

## Abstract

**Simple Summary:**

The objective of this study was to evaluate the effects of supplementation of dairy cows’ diets with different fatty acid (FA) sources on milk production, milk composition, milk fatty acid profile, and physicochemical and sensory characteristics of ice cream. Supplementation (3% dry matter (DM)) of diets with soybean oil (SO) and fish oil (FO) did not have detrimental effects on milk production, milk composition, or ice cream physicochemical and sensory characteristics. From a human standpoint, SO and FO improved the FA profile of milk.

**Abstract:**

The objective of this study was to evaluate the effects of supplementation of dairy cows with different fatty acid sources (soybean oil (SO) and fish oil (FO)) on milk production, milk composition, milk fatty acid profile, and physicochemical and sensory characteristics of ice cream. During 63 days, fifteen Holstein cows averaging 198 ± 35 days in milk were assigned to three groups: control diet with no added lipid (*n* = 5 cows); and supplemented diets with SO (*n* = 5 cows; unrefined SO; 30 g/kg DM) or FO (*n* = 5 cows; FO from unrefined salmon oil; 30 g/kg DM). Milk production, milk fat, and milk protein were not affected by treatments. Saturated fatty acids in milk fat were decreased with SO and FO compared with control. C18:2 *cis*-9, *cis*-12 was increased with SO whereas C18:2 *cis*-9, *trans*-11, C20:3n-3, C20:3n-6, C20:5n-3, and C22:6n-3 were the highest with FO. Draw temperature and firmness were higher in SO compared to control and FO ice creams. Melting resistance was higher in FO compared with control and SO ice creams. Supplementation of cow diets with SO and FO did not have detrimental effects on milk production, or ice cream physicochemical and sensory characteristics.

## 1. Introduction

Despite the fact that dietary guidelines recommend limiting the intake of saturated fatty acids (FA) due to their relation with risk factors for coronary heart disease [1], recent literature has discussed that there is no relation, or a fairly minor association, between saturated FA (SFA) and an increased risk of cardiovascular disease [2]. Milk fat is a considerable source of SFA in human diets [3]. Due to the increased health awareness of consumers, public demand for healthy dairy products is growing and consumers are looking for increased contents of bioactive compounds (such as omega 3 FA) in their purchase choices [4].

The FA profile of milk can be substantially modulated by supplementing animals with different unsaturated FA sources, such as vegetable and marine oils. The addition of vegetable oils in dairy cow diets can increase milk contents of oleic acid (C18:1 *cis*-9), vaccenic acid (C18:1 *trans*-11), linoleic acid (C18:2 *cis*-9, *cis*-12), and rumenic acid (C18:2 *cis*-9, *trans*-11) [5,6], whereas eicosapentanoic acid (EPA; C20:5n-3) and docosahexaenoic acid (DHA; C22:6n-3; [7,8] can be increased when animals are fed with fish oil.

Ice cream is a complex colloid, containing fat globules, air cells, and ice crystals, dispersed in a solution of proteins, polysaccharides, and sugars [9]. Fat plays a critical role in the structure and stabilization of the air phase, and sensory properties of ice cream [10]. Ice cream consumption is a “ludic” activity where most consumers will not compromise enjoyment for the sake of improved nutrition. Therefore, it is necessary for the dairy industry to offer a wide range of products, such as those with lower saturated FA contents and/or bioactive FA that are, perhaps, more suited to regular consumption [11].

Reducing saturated FA and increasing unsaturated FA contents in ice cream may lead to differences in the degree of oxidation or hydrolytic rancidity [12], as well as melting resistance and overrun [13]. In this regard, very little research is available on ice cream manufacturing using milk with different unsaturation degrees of FA. Therefore, the objective of this study was to determine the effect of supplementing cows with different unsaturated FA sources on milk production, milk composition, milk fatty acid profile, and the physicochemical and sensory characteristics of ice cream. The main hypothesis tested in this study was that the degree of unsaturation of dietary lipids (soybean oil as a rich source of C18:2 *cis*-9, *cis*-12 vs. fish oil as a rich source of EPA and DHA) could affect the FA profile of milk and thereby affect the physicochemical and sensory characteristics of ice cream.

## 2. Materials and Methods

### 2.1. Animals and Treatments

Animal care and procedures were carried out according to the guidelines of the animal care committee of the Pontificia Universidad Católica de Chile (project code 150730013). The study was conducted at the Estación Experimental Pirque (33°38’28” S, 70°34’27” W) of the Pontificia Universidad Católica de Chile. Cows were housed in a free-stall barn and fed using Calan Broadbent feeding doors (American Calan, Inc., Northwood, NH, USA) and had continuous access to water.

Fifteen Holstein cows, averaging 198 ± 35 days in milk and a live weight of 637 ± 68 kg at the beginning of the study, were assigned to three treatment groups based on body condition score (BCS; scored on a five-point scale where 1 = emaciated to 5 = obese) and milk yield to achieve comparable groups. Before commencing the study, average BCS for the 3 groups were 2.8 ± 0.3, 2.6 ± 0.2, and 2.7 ± 0.3. Milk yield for the 3 groups was: 40 ± 6, 40 ± 9, and 40 ± 8 kg/d.

Three diets containing 63% forage and 37% concentrate were formulated to meet the nutrient requirements [14] of a 650 kg dairy cow in mid-lactation, eating 26.5 kg of DM per day (Table 1). The control diet contained no added lipid. Diet SO was supplemented with unrefined soybean oil at 30 g/kg of DM. Diet FO was supplemented with unrefined salmon oil at 30 g/kg of DM. Five cows were fed on each diet for a period of 9 weeks. Diets were offered to cows individually, at a fixed daily allowance to ensure complete consumption. Supplementary oils were mixed manually and individually into the diet of cows receiving SO or FO. The most abundant FA (g/100 g of total FA) detected in soybean oil were: C16:0 (14), C18:1 *cis*-9 (17), and C18:2 *cis*-9, *cis*-12 (50). The main FA in fish oil were C16:0 (16), C18:2 *cis*-9, *cis*-12 (16), C20:5n-3 (16), C20:5n-3 (5), and C22:6n-3 (8). Treatment diets were sampled every 14 days and stored at −20 °C for later chemical analyses. Standard procedures for chemical composition analysis of experimental diets were reported previously [5,8]. Ingredients, chemical composition, and FA profile of the diets are shown in Table 1. BCS and body weight were measured on days 21, 42, and 63.

### 2.2. Milk Yield and Composition

Cows were milked three times per day at 07:00, 15:00, and 22:00 h. Milk yield was recorded automatically (DelPro™ FarmManager; DeLaval, Sweden) at each milking. Milk samples were collected from each cow on days 21, 42, and 63, as described by [6], and analysed for fat, protein, and somatic cell count (SCC) with an infrared milk analyser (Milko-Scan CombiFoss 6000; Foss Electric, Hillerød, Denmark).

### 2.3. Fatty Acid Analysis

Milk fat separation was performed with a non-solvent method and the transesterification of FA according to our previous report [6]. A gas chromatograph (GC 2010) system (Shimadzu Scientific Instruments AOC-20s, Columbia, MD, USA) equipped with a 100 m column (Restek Rtx^®^ GC 100 m × 0.32 mm i.d., 0.20 µm film thickness) was used. The GC conditions are reported previously by Vargas-Bello-Pérez et al. [6]. Fatty acid GC peaks were identified by using a FA methyl ester standard (FAME; Supelco 37 Component FAME mix, Bellefonte, PA, USA), and reference standards for C18:1 *trans*-11, C18:1 *cis*-9, *trans*-11, C20:5n-3, and C22:6n-3 (Nu-Chek-Prep Inc., Elysian, MN, USA). Atherogenic index (AI) and thrombogenic index (TI) were calculated according to equations of Ulbricht and Southgate [15].

### 2.4. Ice Cream Manufacturing

Milk collected on days 21, 42, and 63 from cows on the same treatment was pooled and made into ice creams. Raw milks at 45 °C were separated into cream (35% fat) and skim milk (0.1% fat) using a cream separator (Motop Electric, Motor Sich, Zaporozhye, Ukraine). Experimental creams were standardized to a fat content of 19.7%, pasteurized at 85 °C × 5 min, and cooled to 70 °C. The standardized creams (814 g) were mixed with sucrose (170 g; IANSA, Santiago, Chile), vanilla extract (13 g; Good Food S.A., Santiago, Chile), soy lecithin as emulsifier (2 g), and guar gum as stabilizer (1 g; Cherry Chile Ltd.a., Santiago, Chile) to obtain a mixture with 16% fat [16]. The ice cream mixtures were homogenized at 10,000/s for 90 s with an Ultra-Turrax homogenizer (T25; IKA-Labortechnik, Staufen, Germany), cooled to 4 °C with an ice bath, and ripened at 4 °C for 24 h. The manufacturing of ice cream was performed on a 900 g scale using a batch ice cream maker (Blanik BICM055M with 1.4 L of capacity; Blanik SPA, Santiago, Chile) under constant stirring for 30 min. The samples were then hardened at −20 °C for 48 h for further analyses.

### 2.5. Physical Properties of Ice Cream

The temperature of the obtained soft ice-creams was recorded (immediately after manufacturing) and reported as draw temperature [10]. The overrun of experimental ice creams was measured based on the proportion of weight of soft ice cream to the weight of ice cream mixture, as described by Adapa et al. [17].

A penetration test was performed on ice cream samples using a texture analyzer (TA-XTi2 Stable Micro Systems Co., Ltd., Surrey, UK) equipped with a 30 kg loading cell. Prior to analysis, 50 g of soft ice cream samples were placed in 70 mL plastic cups (42 mm height, 35 mm lower diameter, and 60 mm top diameter) and hardened for 48 h at −20 °C. Samples were then penetrated for 15 mm at the geometrical center at a rate of 0.5 mm/s. The firmness (N) was expressed as the peak force obtained during testing. Five ice cream samples were analyzed per treatment.

The melting properties of ice creams were estimated at 20 °C on 30 g of ice creams positioned on a 0.1 mm wire mesh above a pre-weighed 100 mL beaker. The melting rate was estimated based on the slope obtained from weighting the beaker at 5 min intervals during 45 min after the first dripping [18].

The color of experimental ice cream at −20 °C was recorded with a Konika-Minolta colorimeter CR-400 (Konica-Minolta Optics Inc., Osaka, Japan) on the surface of hardened samples in plastic cups. The instrument was set on the CIELAB system, an illuminant D65, and a visual angle of 2°. In addition, the whiteness index (WI) was also estimated from color measurements [18].

### 2.6. Sensory Analysis of Ice Cream

The sensory properties of experimental ice creams were evaluated by using a consumer’s test. The consumers were recruited based on their interest and availability to participate the research. They all consumed ice cream at least twice a week. Judges (20) were not provided with any information regarding treatment of samples in any testing session.

Ice cream samples were identified with a random 3-digit code. Thirty grams of ice cream samples were served in plastic cups and evaluated with a 1–9 hedonic scale (1 = extremely dislike; 5 = neither like nor dislike; 9 = extremely like). The evaluated attributes were appearance, texture, melting resistance, taste, aroma, milkfat, and general acceptability. The panelists were also requested to write additional comments on the evaluation form [19].

### 2.7. Statistical Analyses

Data were analyzed using linear mixed models fitted with PROC MIXED in SAS (SAS Institute Inc., Cary, NC, USA). Fixed effects in the models were diet, time, and the diet × time interaction. Random effects in the models were cow within diet. Option PDIFF (Piecewise Differentiable) was used to test differences between least squares means for fixed effects. Models were fitted for: milk yield, composition, and FA; ice cream composition, FA, physical characteristics, and sensory evaluations; body weight; and BCS. Data for SCC were log-transformed before analysis, but results are presented as back-transformed least squares means and errors.

## 3. Results and Discussion

### 3.1. Diets and Animal Performance

Dietary treatments reflected the FA composition (g/100 g of total FA) of supplemented oils (Table 1). Except for a slight change (*p* < 0.05) in protein contents (higher in SO), treatments did not affect animal performance and milk composition (Table 2). Those results agree with the lack of differences on performance and milk composition in previous reports, where cows were fed with 4.0 or 2.6% DM of SO ([5,20], respectively), or with 2.6 or 2.7% DM of FO ([7,8], respectively). In this experiment, a moderate quantity of oil supplementation was used, and it was sufficient to alter the milk FA profile and ice cream physicochemical characteristics. However, it is known that when the inclusion of dietary oil is 5% of DM or more, dry matter intake depression or milk fat depression is most likely observed [21].

Milk protein content (g/100 g) was higher (*p* < 0.05) in SO compared with control and FO. SO possibly improved the energy density required for microbial protein synthesis and this was reflected in increased milk protein contents [22]. Production and milk composition parameters did not have diet × time interactions. Treatment did not affect milk SCC. High SCC of some individual cows influenced treatment means, and the standard error of the mean, but these cows did not show abnormal values for other constituents.

Milk yield was decreased (*p* < 0.05) during the experiment from 43.2 to 42.4 kg/d. One explanation to this effect is that the animals used for the study were normally managed in groups of animals of approximately 70 cows in dry lot systems. For this study, animals were confined in a pen and selected from different productive herds. Broucek et al., [23] reported that transferring lactating dairy cows to unknown pens with unknown animals might result in signs of stress that include a long-term suppression of milk efficiency and overall production performance.

### 3.2. Milk Fatty Acid Profile

Fatty acids in milk originate from mammary uptake of preformed FA from blood and de novo synthesis in the mammary gland. All C4:0 to C12:0 FA, the majority of C14:0 (ca. 95%), and approximately half of C16:0 in milk fat derives from de novo synthesis, whereas half of C16:0 and all C18 and very-long chain FA originate from feed, ruminal metabolism, and body fat reserves [3]. De novo synthesis in the mammary gland decreases when the availability of these preformed FA increases. The milk fat of dairy cows contains high proportions of SFA and consequently lower proportions of monounsaturated FA (MUFA) and polyunsaturated FA (PUFA), due to ruminal lipolysis and biohydrogenation of dietary unsaturated fatty acids and de novo synthesis [3]. In addition, the PUFA absorbed in the small intestine are incorporated in phospholipids and cholesterol esters that are not as readily available FA sources for the mammary gland as FA in triacylglycerols [24].

The fatty acid composition of milks is shown in Table 3. Compared with control and SO, C4:0, C14:1 *cis*-9, C18:1 *trans*-11, C18:1 *cis*-9, C18:2 *cis*-9, *trans*-11, C20:5n-3, C24:1n-9, and C22:6n-3 were higher (*p* < 0.05) in FO. SO increased (*p* < 0.05) C18:2 *cis*-9, *cis*-12, and C18:3 *cis*-9, *cis*-12, and *cis*-15 in milk fat compared to control and FO. Both SO and FO increased (*p* < 0.05) C18:0, and decreased (*p* < 0.05) C6:0, C8:0, C10:0, and C14:0 compared with control. The most abundant FA in milk fat across all diets was C16:0, followed by C18:0 and C18:1 *cis*-9. C18:1 *cis*-9, the most abundant *cis*-MUFA in milk fat, originates directly from the ruminal escape of C18:1 *cis*-9 as well as Δ^9^ desaturation of C18:0 in the mammary gland. Desaturation of C18:0 is responsible for 60% of C18:1 *cis*-9 in milk, and in ruminants, around 40% of C18:0 taken up by the mammary gland is desaturated to C18:1 *cis*-9 by the Δ^9^ desaturase enzyme [25]. In this study, C18:0 and C18:1 *cis*-9 were increased (*p* < 0.05) by both SO and FO compared with control. The results regarding the effects of SO diet on milk FA composition were as expected, as plant oil supplements and oilseeds enriched with C18:2 *cis*-9, *cis*-12 in dairy cow feeding typically increase the availability of C18:0 for the mammary gland and elevate *cis*-MUFA levels (mainly C18:1 *cis*-9) in milk fat [3]. However, we observed an increase (*p* < 0.05) in both C18:0 and C18:1 *cis*-9 in milk fat from FO diet, although marine oil supplementation in dairy cow diets usually decreases both of these FA in milk fat [26]. In humans, dietary C18:1 *cis*-9 can lower total blood cholesterol and low-density lipoprotein [27].

Both SO and FO increased (*p* < 0.05) levels of C18:2 *cis*-9, *trans*-11 when compared with control, but FO diet resulted in the highest (*p* < 0.05) proportions which is in line with the results from earlier studies. Plant oils containing abundant amounts of C18:2 *cis*-9, *cis*-12, and C18:3 *cis*-9, *cis*-12, *cis*-15 increase C18:2 *cis*-9, *trans*-11 concentrations in milk fat [3]. C18:2 *cis*-9, *trans*-11 in milk fat originates directly from ruminal biohydrogenation of C18:2 *cis*-9, *cis*-12, and C18:3 *cis*-9, *cis*-12, *cis*-15 and from increased outflow of C18:1 *trans*-11, a biohydrogenation intermediate, which is further desaturated in the mammary gland by Δ^9^ desaturase [28]. In accordance with previous studies [3], FO high in n-3 PUFA induced higher proportions of C18:2 *cis*-9, *trans*-11 in milk fat compared with SO. It has been reported that supplementation of moderate amounts of FO as Ca salt (2.7% DM; [7] or as unrefined salmon oil (2.6% DM; [5] in dairy cow diets increases the contents of C18:1 *trans*-11 and C18:2 *cis*-9, *trans*-11 in milk FA. This is because the very long-chain n-3 PUFA in marine lipid supplements inhibit the last biohydrogenation step of 18-carbon FA, i.e., the conversion of C18:1 *trans*-11 to C18:0, resulting in accumulation of C18:1 *trans*-11 in the rumen, which provides more C18:1 *trans*-11 to the mammary gland that can be converted to C18:2 *cis*-9, *trans*-11 by the Δ^9^ desaturase [24].

Because the transfer rate of C20:5n-3 and C22:6n-3 into milk is extremely limited even in high levels of supplementation due to their extensive ruminal biohydrogenation and utilization in other functions than milk fat synthesis, marine lipid supplements in dairy cow feeding are mainly studied for their role as inhibitors of the conversion of C18:1 *trans*-11 to C18:0 in the rumen [3,26]. In addition, fish oil and marine algae supplements may decrease milk fat synthesis, especially if fed in high amounts to dairy cows [3,26]. Nevertheless, C20:3n-3, C20:5n-3, C22:6n-3, and total omega-3 (n-3) FA were higher (*p* < 0.05) in milk from FO treatment compared with control and SO (Table 4). Higher n-3 levels in blood or on the erythrocyte membrane are associated with reduced cardiovascular mortality [29]. Even though C20:5n-3 and C22:6n-3 were increased in FO milk, their concentrations were not enough to markedly contribute to the human intake recommendations (approx. 1 g/d) of n-3 to reduce the risk of cardiovascular disease [30]. Even though the n-3 levels were increased in FO, the total PUFA were not changed relative to control. However, the total PUFA in milk fat from SO diet were increased (*p* < 0.05) compared with other diets. This reflects the higher intake and ruminal escape of C18:2 *cis*-9, *cis*-12 in SO, although transfer efficiency of C18:2 *cis*-9, *cis*-12 from plant oils and oilseed supplements to milk is low, as it is for the very long-chain PUFA [3].

There were some time effects which may reflect possible rumen microbial adaptations to the oil treatments [31]. In some cases, SO and FO decreased individual SFA and increased some unsaturated FA. For example, from 21 to 63 days of supplementation with SO and FO, the following milk FA were decreased: C6:0, C14:0, C16:0, and C18:0, whereas C14:1 *cis*-9, C18:1 *trans*-10, C18:1 *trans*-11, C18:1 *cis*-9, C18:3 *cis*-6, *cis*-9, and *cis*-12 were increased. Interestingly, with the exception of C20:0 and C24:0, FA with more than 20 carbons had a quadratic effect: they increased from day 21 to 42 and then decreased in day 63 with FO.

Main fatty acid groups in milk fat, calculated fatty acid ratios, health lipid indices, and product/substrate concentration ratios as estimates for Δ^9^ desaturase activity in the mammary gland are shown in Table 4. In general, SFA concentrations of milk were reduced (*p* < 0.05) with SO and FO treatments compared with control. According to Ulbricht and Southgate (1991), C12:0, C14:0, and C16:0 are SFA that can promote atherosclerosis and coronary thrombosis. The reduction of SFA content in milk has been reported previously when plant oils such as SO [3] or FO alone [3,5,7], or in combination with vegetable oils [5,24], are incorporated into dairy cow diets. As dairy products are a major source of C12:0, C14:0, and C16:0 in the human diet [3], the reduction in the level of 14:0 in milk fat from cows fed SO and C14:0 and C16:0 in milk fat of cows fed FO in this study is a favourable change. Contrary to the previous studies with SO or other plant oils [3], SO supplement did not induce a reduction in the proportion of C12:0 nor C16:0 in the present study. Generally, according to the comprehensive literature review of Kliem and Shingfield [3], fish oil and marine algae induce more subtle changes in C12:0, C14:0, and C16:0 proportions in milk fat compared with vegetable oils, and marine lipids generally cause a decrease in the proportion of 18:0, whereas vegetable oils elevate its levels in milk fat. Thus, the results of the present study regarding changes induced by FO are different than expected, but more favourable to human health. Naturally, decreases in the levels of SFA in milk fat are accompanied by increases in unsaturated FA. For the SO diet, the total PUFA in milk fat increased compared with control, whereas the FO diet induced increases in MUFA. When dietary SFA were replaced with *cis*-unsaturated FA, the level of serum lipid biomarkers were improved, thus reducing risk factors of CVD [32].

Total SFA, PUFA, n-3 PUFA, n-6/n-3, atherogenicity index, and thrombogenicity index were decreased (*p* < 0.05) from day 21 to 63 by SO and FO. These results reflected findings from individual milk FA as they are used for the calculation of the aforementioned groups of lipids, ratios, or indices. The ratio C14:1 *cis*-9/C14:0 was higher (*p* < 0.05) in FO compared with control and SO. The C14:0 is synthesized in the mammary gland, and therefore C14:1 *cis*-9 can only be produced by desaturation through Δ9-desaturase enzyme. The average Δ9-desaturase activity for C14:1 *cis*-9/C14:0 was 0.03 in this study. However, Bu et al. [20] reported that this index ranges from 0.048 to 0.085 depending on the fat supplement.

The increases observed in milk C18:2 *cis*-9, *trans*-11 can be partially attributed to the increased index of C18:2 *cis*-9, *trans*-11/C18:1 *trans*-11 in the FO and SO over the control (Table 4). Increasing contents of C18:2 *cis*-9, *trans*-11 (also known as rumenic acid) is desirable in dairy products. Human studies have reported the health advantages of consuming C18:2 *cis*-9, *trans*-11, since it might improve cancer prevention, cardiovascular diseases, immune and inflammatory responses, and bone health [33]. It should be noted that the primary dietary sources of C18:2 *cis*-9, *trans*-11 for humans are dairy products and meat products from ruminant animals.

### 3.3. Physicochemical Properties of Ice Cream

This study focused on the interface of animal production and food science, and thus, our approach was to improve milk FA profile for ice cream production by modifying the cow’s diet.

In order to analyze the effect of dietary lipids, all ice cream treatments were standardized to obtain a final fat content of 16%. It has been reported that a reduction in the fat content in the ice cream particularly to or below 30 g/kg can result in the loss of textural and sensory properties [34]. The protein content of ice cream was slightly higher (*p* < 0.05) in SO compared with control, whereas FO did not differ from other treatments. However, those changes were minimal (<0.2%), and therefore they were unlikely to affect the overall nutritional and functional properties of ice cream. Fat, lactose, and sucrose were not affected by treatments and there was not treatment × time interactions (Table 5).

Physical parameters of ice creams are shown in Table 5. Draw temperature was higher (*p* < 0.05) in control and SO compared with FO. The lower draw temperature in FO ice creams could be associated with their higher contents of monounsaturated FA in the milk used for ice cream manufacturing. Overrun was lowered in FO ice creams compared with control. Ice creams that have more saturated FA may form solidified fat at a higher temperature during ice cream manufacturing, leading to a higher efficiency on retaining air in the matrix [35], which is reflected in increased overrun in the control and SO ice creams.

Firmness was similar in control and SO but lower (*p* < 0.05) in FO. Melting rate was higher (*p* < 0.05) in FO compared to control and SO. Firmness and melting rate results can be explained because FO had an increased proportion of unsaturated FA compared with control and SO that may have more liquid-like matrix behaviour, in contrast with saturated FA which will have a more solid-like form at low temperatures [36]. Firmness of ice cream can be influenced by numerous factors such as ice crystal content, ice crystal size, extent of fat destabilization, overrun, and the rheological properties of the mix [37]. Studying the above-mentioned factors would be highly recommended in future experiments where milks with high-unsaturated FA profile for ice cream manufacturing are used.

The lowered (*p* < 0.05) yellowness (b *) intensity found in SO and FO may be explained by the FA unsaturation that those ice creams had. Previously, it has been reported that the b value increases as the fat content of samples increases [38].

### 3.4. Sensory Properties of Ice Cream

Despite the fact that more than 50% of FA were numerically different in the milks used for ice cream manufacturing, the sensory properties of ice creams were not significantly different (*p* > 0.05) between treatments (Table 6). However, six of the panellists noted that there was an off-flavor from FO ice creams. This could be an indicator of lipid oxidation that often occurs when unsaturated FA matrices are subjected to intense heat treatments. In this study, high pasteurization treatment (85 °C × 5 min) applied to inhibit lipase activity in standardized creams could have generated lipid oxidation [39] that was detected by some panellists.

Panellists judging milks [40] and cheeses [5,7] from cows fed on FO have noted off-flavors such as metallic and oxidized flavors. However, in those previous studies, the odd flavors are often noted by few panellists and compared to their control treatments; changes are minimum and never reach statistical significance.

From a flavor perspective, malonaldehyde and 1-octen-3-one are important products of lipid oxidation [41]. In this study, detection of off-flavors may be due to the presence of lipid oxidation products such as 1-octen-3-one, which has a metallic flavor and is a key component in many oxidized flavors. Malonaldehyde is a component without flavor, but its measurement through thiobarbituric acid tests has been a means of detecting oxidation and oxidized flavors. Further research should consider analyzing these lipid oxidation products to complement sensory analysis.

## 4. Conclusions

Results showed that supplementation (3% DM) of dairy cow diets with SO and FO did not have detrimental effects on milk production, or ice cream’s physicochemical and sensory characteristics. From a human standpoint, SO and FO improved the FA profile of milk by increasing the amounts of PUFA and MUFA, respectively. From a farmer perspective, adding SO or FO in the cow’s diet can improve the milk FA profile towards a more unsaturated one (without detrimental effects on cows’ performance) and this can lead for the production of niche products with bioactive lipids. Data from this study could be useful for the dairy industry, especially for the ice cream industry that is exploring or looking to provide an added value for their products via natural means (cows diet modulation) and without removing fat contents.

## Figures and Tables

**Table 1:** Ingredient and chemical composition of control, soybean oil (SO), and fish oil (FO) dietary treatments.

Component	Diet		
	Control	SO	FO
Ingredient composition (% DM)			
Corn silage	32.0	31.1	31.1
Fresh alfalfa	24.0	23.3	23.3
Malt distillers	19.2	18.6	18.6
Corn grain	7.6	7.4	7.4
Canola meal	6.2	6.0	6.0
Alfalfa hay	5.0	4.9	4.9
Soybean grain	4.0	3.9	3.9
Wheat bran	1.6	1.6	1.6
Vitamin and mineral premix ^1^	0.4	0.4	0.4
Soybean oil	0	2.9	0
Fish oil	0	0	2.9
Chemical composition (% DM)			
Dry matter	42.5	44.3	43.8
Crude protein	13.7	12.9	13.0
Ether extract	3.4	7.6	7.4
Neural detergent fibre	29.9	29.8	28.9
Acid detergent fibre	17.3	17.5	17.7
Lignin	3.5	3.6	3.5
Ash	7.2	6.8	6.8
Fatty acid composition (g/100 g of FA)			
C6:0	0.93	0.1	0.1
C10:0	0.25	nd	nd
C12:0	1.13	0.2	0.1
C14:0	10.35	0.6	7.05
C15:0	5.44	nd	4.06
C16:0	6.72	13.79	16.14
C16:1 *cis*-9	nd ^2^	1.7	4.53
C17:0	1.29	0.97	1.05
C18:0	22.55	5.17	8.72
C18:1 *cis*-9	0.92	17.94	7.94
C18:2 *cis*-9, *cis*-12	33.73	49.89	16.07
C18:3 *cis*-6, *cis*-9, *cis*-12	7.72	2.83	2.63
C18:3 *cis*-9, *cis*-12, *cis*-15	8.97	6.81	3.25
C20:5n-3	nd ^2^	nd	15.62
C22:5n-3	nd	nd	4.79
C22:6n-3	nd	nd	7.95

^1^ Contained per kg: 25 g of P; 80 g of Ca; 25 g of Mg; 1.6 g of S; 300,000 IU of vitamin A; 50,000 IU of vitamin D_3_ and 1600 IU of vitamin E; ^2^ nd = not detected.

**Table 2:** Performance and proximate analysis of milk from cows supplemented with control, soybean oil (SO), and fish oil (FO) ^1^.

Parameter	Diet ^2^			SEM	*p*-Value	
	Control	SO	FO		Diet	Time ^3^
Production						
Dry matter intake (kg/DM day)	26.5	26.5	26.5	*	*	*
Milk yield, kg/day	43.2	42.4	43.2	1.5	0.966	<0.001
Fat, kg/day	1.61	1.51	1.43	0.10	0.430	0.750
Protein, kg/day	1.47	1.53	1.50	0.06	0.800	0.650
Body weight, kg	633	640	628	15	0.966	0.033
Body condition score ^4^	2.55	2.61	2.48	0.08	0.178	0.792
Milk composition, g/100 g						
Fat	3.72	3.53	3.31	0.24	0.493	0.236
Protein	3.40 ^b^	3.59 ^a^	3.45 ^b^	0.05	0.040	0.425
Urea (mg/100 mL)	34.5	27.6	32.4	2.4	0.120	0.350
Somatic cell count, × 10^3^/mL	85	382	481	269	0.520	0.577

^a,b^ Means in the same row with different superscripts are different (Diet *p* < 0.05); ^1^ Values are LSM and pooled SEM, *n* = 45; ^2^ Control = No fat supplement; SO = supplement of 30 g/kg DM; FO = supplement of 30 g/kg DM; ^3^ Time effect over 21, 42, and 63 days; ^4^ BCS = Scored on a five-point scale where 1 = emaciated to 5 = overly fat. There was not diet × time interactions. * Cows were individually fed at a fixed rate and did not show feed refusals.

**Table 3:** Milk fatty acid profile from cows supplemented with control, soybean oil (SO), and fish oil (FO) ^1^.

Fatty Acid (g/100 g of FA)	Diets ^2^			SEM	*p*-Value		
	Control	SO	FO		Diet (D)	Time (T) ^3^	D × T
C4:0	2.27 ^b^	2.22 ^b^	2.34 ^a^	0.19	<0.001	0.084	0.031
C6:0	1.75 ^a^	1.15 ^b^	1.49 ^b^	0.12	<0.001	0.006	0.196
C8:0	1.34 ^a^	0.78 ^b^	0.88 ^b^	0.16	0.002	0.240	0.754
C10:0	2.11 ^a^	1.79 ^b^	1.69 ^b^	0.16	0.031	0.266	<0.001
C11:0	0.32	0.19	0.32	0.10	0.320	0.582	0.018
C12:0	2.10	2.11	2.36	0.17	0.265	<0.001	<0.001
C13:0	0.13	0.12	0.15	0.07	0.895	0.332	0.023
C14:0	11.89 ^a^	8.42 ^b^	9.97 ^b^	0.40	<0.001	<0.001	<0.001
C14:1 *cis*-9	0.58 ^b^	0.34 ^c^	0.99 ^a^	0.12	<0.001	<0.001	0.056
C15:0	0.41	0.37	0.38	0.11	0.901	0.333	0.007
C15:1 *cis*-9	0.76	0.42	0.70	0.10	0.005	0.056	0.540
C16:0	31.26 ^a^	32.79 ^a^	28.92 ^b^	0.69	<0.001	<0.001	<0.001
C16:1 *cis*-9	1.21 ^a^	0.66 ^b^	1.36 ^a^	0.12	<0.001	0.588	0.012
C17:0	0.34	0.35	0.36	0.17	0.994	0.459	0.298
C17:1 *cis*-9	0.34	0.24	0.30	0.04	0.011	0.308	0.982
C18:0	19.55 ^b^	20.95 ^a^	22.03 ^a^	0.78	0.011	<0.001	<0.001
C18:1 *trans*-10	0.37	0.31	0.39	0.11	0.759	<0.001	0.576
C18:1 *trans*-11	0.15 ^c^	0.19 ^b^	0.26 ^a^	0.03	0.090	<0.001	0.012
C18:1 *cis*-9	17.46 ^c^	18.90 ^b^	19.05 ^a^	0.76	0.041	<0.001	<0.001
C18:2 *cis*-9, *cis*-12	0.66 ^b^	1.86 ^a^	0.40 ^b^	0.18	<0.001	0.169	<0.01
C18:2 *trans*-9, *trans*-12	0.73 ^b^	0.63 ^b^	1.00 ^a^	0.10	<0.001	0.580	0.044
C18:3 *cis*-6, *cis*-9, *cis*-12	0.51	0.69	0.46	0.15	0.304	0.044	0.030
C18:3 *cis*-9, *cis*-12, *cis*-15	0.20 ^b^	0.61 ^a^	0.33 ^b^	0.13	<0.001	<0.001	<0.001
C18:2 *cis*-9, *trans*-11	0.50 ^c^	1.18 ^b^	1.75 ^a^	0.17	<0.001	0.048	<0.001
C20:0	0.08	0.17	0.08	0.06	0.234	0.393	0.278
C20:1n-9	0.06	0.08	0.03	0.03	0.411	0.014	0.122
C20:2	0.02 ^b^	nd ^4^	0.04 ^a^	0.03	0.024	0.029	0.343
C22:0	0.08	0.04	0.04	0.03	0.392	0.014	0.540
C20:3n-3	0.19 ^b^	0.12 ^c^	0.34 ^a^	0.05	<0.001	<0.001	0.045
C20:3n-6	0.20 ^b^	0.17^b^	0.29 ^a^	0.04	0.026	<0.001	0.096
C22:1n-9	0.01 ^b^	nd	0.05 ^a^	0.02	0.010	0.017	0.006
C23:0	0.02	0.05	0.03	0.03	0.525	0.014	0.044
C20:4n-6	0.04 ^b^	0.11 ^a^	0.11 ^a^	0.03	0.018	0.010	<0.001
C22:2	0.02 ^b^	nd	0.04 ^a^	0.01	0.024	0.029	0.343
C24:0	0.01	0.02	0.02	0.01	0.737	0.560	0.075
C20:5n-3	0.11 ^b^	0.11 ^b^	1.17 ^a^	0.07	<0.001	<0.001	<0.001
C24:1n-9	0.07 ^b^	0.10 ^b^	0.56 ^a^	0.06	<0.001	0.005	<0.001
C22:6n-3	0.07 ^b^	0.06 ^b^	0.41 ^a^	0.05	<0.001	<0.001	<0.001

^a,b,c^ Means in the same row with different superscript letters are significantly different (Diet *p* < 0.05); ^1^ Values are LSM and pooled SEM, *n* = 45; ^2^ Control = No fat supplement; SO = supplement of 30 g/kg DM; FO = supplement of 30 g/kg DM; ^3^ Time effect over 21, 42, and 63 days; ^4^ nd: not detected.

**Table 4:** Groups of main fatty acids, fatty acid ratios, health lipid indices, and estimated Δ^9^-desaturase activity of milk fatty acids in the mammary gland from cows supplemented with control, soybean oil (SO), and fish oil (FO) ^1^.

Fatty Acids (g/100 g of FA)	Diets ^2^				*p*-Value		
	Control	SO	FO	SEM	Diet (D)	Time (T) ^3^	D × T
Σ Saturated fatty acids	74.75 ^a^	71.49 ^b^	71.06 ^b^	0.78	<0.001	<0.001	<0.001
Σ Monounsaturated fatty acids	21.08 ^b^	21.21 ^b^	23.69 ^a^	0.80	<0.001	<0.001	<0.001
Σ Polyunsaturated fatty acids	4.16 ^b^	7.30 ^a^	5.25 ^b^	0.37	<0.001	0.031	<0.001
Σ n-6 polyunsaturated fatty acids	2.15 ^b^	3.46 ^a^	2.27 ^b^	0.26	<0.001	0.258	<0.001
Σ n-3 polyunsaturated fatty acids	0.69 ^c^	2.01 ^b^	2.40 ^a^	0.16	<0.001	<0.001	<0.001
Indices							
n-6/n-3	3.82 ^a^	1.88 ^b^	1.36 ^b^	0.33	<0.001	0.232	<0.001
PUFA/SFA	0.06 ^b^	0.10 ^a^	0.07 ^b^	0.01	<0.001	<0.001	<0.001
Atherogenicity index	1.55 ^a^	0.87 ^c^	1.22 ^b^	0.06	<0.001	0.016	<0.001
Thrombogenicity index	2.67 ^a^	2.13 ^b^	1.96 ^c^	0.07	<0.001	<0.001	<0.001
C14:1 *cis*-9/C14:0	0.05 ^b^	0.04 ^b^	0.10 ^a^	0.01	<0.001	<0.001	0.053
C18:2 *cis*-9, *trans*-11/ C18:1 *trans*-11	0.03 ^b^	0.09 ^a^	0.09 ^a^	0.01	<0.001	0.032	<0.001

^a,b,c^ Means in the same row with different superscript letters are significantly different (Diet *p* < 0.05); ^1^ Values are LSM and pooled SEM, *n* = 45; ^2^ Control = No fat supplement; SO = supplement of 30 g/kg DM; FO = supplement of 30 g/kg DM; ^3^ Time effect over 21, 42, and 63 days.

**Table 5:** Calculated composition and physical properties of ice creams from cows supplemented with control, soybean oil (SO), and fish oil (FO) ^1^.

Item	Diet ^2^			SEM	*p*-Value	
	Control	SO	FO		Diet	Time ^3^
Fat, g/100 g	16.15	16.15	16.14	<0.01	0.518	0.754
Protein, g/100 g	2.78 ^b^	2.93 ^a^	2.82 ^ab^	0.03	0.030	0.630
Lactose, g/100 g	3.99	3.99	3.98	<0.01	0.325	<0.001
Sucrose, g/100 g	17.09	17.09	17.10	0.01	0.780	0.383
Draw temperature (°C)	−4.90 ^a^	−5.17 ^a^	−4.10 ^b^	0.15	0.016	0.229
Overrun (%)	8.02 ^a^	5.10 ^ab^	3.08 ^b^	0.60	0.011	0.708
Firmness (N)	15.90 ^a^	15.55 ^a^	12.59 ^b^	0.44	0.011	0.370
*Melting*						
Dripping time (min)	53.20	68.17	60.17	4.13	0.143	0.022
Melting rate (g/min)	0.62 ^b^	0.87 ^ab^	0.94 ^a^	0.08	0.037	0.666
*CIELAB color*						
L *	84.36	88.44	84.97	2.38	0.492	0.526
a *	−1.08	−0.99	−1.17	0.04	0.060	0.642
b *	20.49 ^a^	17.18 ^b^	15.36 ^b^	0.80	0.025	0.987
Whiteness index (WI)	75.69	77.17	78.66	2.05	0.296	0.627

L * (lightness or whiteness; L * = 0 for black and L * = 100 for white color); a * (red-green components, −a * = greenness and +a * = redness); b * (yellow-blue components, −b * = blueness and +b * = yellowness) ^a,b^ Means in the same row with different superscripts are different (Diet *p* < 0.05); ^1^ Values are LSM and pooled SEM, *n* = 9; ^2^ Control = no fat supplement; SO = supplement of 30 g/kg DM; FO = supplement of 30 g/kg DM; ^3^ Time effect over 21, 42, and 63 days. There was no diet × time interaction.

**Table 6:** Sensory properties of ice creams from cows supplemented with control, soybean oil (SO), and fish oil (FO) ^1^.

Attribute	Diet ^2^			SEM	*p*-Value	
	Control	SO	FO		Diet	Time ^3^
Appearance	6.5	6.6	6.6	0.1	0.921	0.904
Texture	6.8	6.7	6.9	0.2	0.606	0.565
Melting resistance	6.1 ^b^	6.3 ^b^	6.8 ^a^	0.1	0.004	0.057
Taste	6.6	6.4	6.4	0.4	0.959	0.416
Aroma	6.1	5.6	5.8	0.2	0.401	0.213
Milk fat	7.0	6.5	7.0	0.2	0.327	0.610
General acceptability	6.7	6.5	6.5	0.4	0.889	0.596

^a,b^ Means in the same row with different superscripts are different (Diet *p* < 0.05); ^1^ Values are LSM and pooled SEM, *n* = 180 (3 replicates × 3 treatments × 20 panelists); ^2^ Control = No fat supplement; SO = supplement of 30 g/kg DM; FO = supplement of 30 g/kg DM; ^3^ Time effect over 21, 42, and 63 days. There was no diet × time interaction.
